# Clarifying the origin of the interpubic cavity using a biomechanical model and the finite element method

**DOI:** 10.1038/s41598-025-22293-8

**Published:** 2025-11-03

**Authors:** Zdeněk Horák, Matěj Mazura, Václav Báča, Miloslav Vilimek, Martin Salášek, Alžběta Blanková, David Kachlík, Robert Grill, Valér Džupa

**Affiliations:** 1 Department of Technical Studies, College of Polytechnics Jihlava, Jihlava, Czech Republic; 2 Department of Pathophysiology, Third Faculty of Medicine, Charles University, Prague, Czech Republic; 3 Center for Endoscopic, Surgical and Clinical Anatomy (CESKA), Second Faculty of Medicine, Charles University, Prague, Czech Republic; 4 First Department of Orthopaedics, First Faculty of Medicine, Charles University and Motol University Hospital, V Úvalu 84, Prague 5, 150 06 Czech Republic; 5 Department of Anatomy, Second Faculty of Medicine, Charles University, Prague, Czech Republic; 6 Department of Anatomy, Third Faculty of Medicine, Charles University, Prague, Czech Republic; 7 Department of Orthopedics and Traumatology, Faculty of Medicine, Charles University, and University Hospital, Pilsen, Czech Republic; 8 New Technologies for the Information Society, Faculty of Applied Sciences, University of West Bohemia, Pilsen, Czech Republic; 9 Department of Histology and Embryology, Third Faculty of Medicine, Charles University, Prague, Czech Republic; 10 Department of Urology, Third Faculty of Medicine, Charles University, University Hospital Kralovske Vinohrady, Prague, Czech Republic; 11 Department of Orthopedics and Traumatology, Third Faculty of Medicine, Charles University, University Hospital Kralovske Vinohrady, Prague, Czech Republic

**Keywords:** Finite element analysis, Pubic symphysis, Interpubic cavity, Anatomy, Musculoskeletal system

## Abstract

The interpubic cavity is a narrow, slit-like, oval-shaped cavity which has frequently been described within the fibrocartilaginous interpubic disc. The aim of the study was to clarify the origin of the primary interpubic cavity. Knowledge of the physiological load of the pubic symphysis while standing and walking is important when deciding on the load after pelvic fractures and when developing implants to fix injuries in the pubic region, and therefore this knowledge could clarify the origin of the interpubic cavity. It is not possible to measure the load on the pubic symphysis in clinical practice, therefore the authors simulated the load on the pelvis during walking using the finite element analysis. Analyses were performed on a physiological pelvis model loaded with a force corresponding to the reaction force in the hip joint. The pubic symphysis was more heavily loaded with forces from muscle groups, the maximum deformation on the pubic symphysis was localised to two places that are permanently loaded during physiological walking. The area associated with the distribution of reduced stress, the middle and caudal parts of the interpubic disc, correlates very well with the frequency of occurrence of the interpubic cavity. It can therefore be assumed that the cavity helps the joint with load bearing during the transition to bipedal walking, i.e., at the time of high biomechanical load. This study will help us clarify the origin of the interpubic cavity.

## Introduction

The interpubic cavity (interpubic cleft; IPC) is a narrow, slit-like, oval-shaped cavity which has frequently been described within the fibrocartilaginous interpubic disc. The interpubic disc is a part of the symphysial joint called the pubic symphysis. Pubic symphysis is a non-synovial joint^1^. A cavity is defined as appearing around the second year postnatally and a secondary cavity as appearing in adulthood^2^. The IPCs resemble a lens in shape and have well defined contours^2–5^. Causes of theIPC forming are unknown and have not been described in any available literature yet. Understanding the physiological loading of the pubic symphysis while standing will help us clarify the origin of the interpubic cavity during the transition to bipedal walking. Direct measurement of the pubic symphysis loads is almost impossible using conventional methods. Therefore, we simulated pelvic loads while walking using the finite element method (FEM). The aim was to monitor the response of a physiological model of the pelvis to external loads, especially around the pubic symphysis. Two situations were evaluated, in which the pelvic model was loaded with (1) only the reactive forces acting on the hip joint while standing on one leg and (2) the individual muscles located in the pelvic region, which are active when standing on one leg. We compared the findings with information about the structure of the pubic symphysis obtained from evaluating MR images.

The aim of the study was to clarify the origin of the interpubic cavity. After finding out the cause of the cavity developemnt, we will be better able to understand the meaning of the cavity and its effect on the function of an important pelvic joint.

## Methods

The finite element method (FEM) was used to evaluate the deformation and stress response of the pelvis model to load. We believe that the FEM allows for Level of Evidence III conclusions. During the analyses, the response of individual parts of the model under external load was monitored in detail. A geometrical model of the pelvis was created from a series of CT images of a healthy female. The images were taken at a resolution of 512 × 512 pixels, with a pixel size of 0.412 mm and a slice thickness of 0.5 mm. CT images were imported as DICOM (Digital Imaging and Communications in Medicine) files into the specialized Mimics program (Materialise, Leuven, Belgium), in which a 3D reconstruction of individual structures was performed. The geometric model of the bones was created using a surface triangular mesh in the STL (Stereolithography) format. The resulting model situation set was imported into theAbaqus program (Dassault Systèmes, Vélizy-Villacoublay, France). A physiological model of the pelvis was analyzed, i.e., without injury or structural change. The model consisted of the left and right hip bones and the pubic symphysis. It was a young adult woman (22 years old) with no known systemic diseases. Anamnesis without pathology that could affect the biological properties of the pelvic ring.

In the computational analyses, the pubic symphysis was modeled using the Mooney-Rivlin hyperelastic material model with material parameters (C_10_ = 0.1 MPa, C_01_ = 0.45 MPa, and C_11_ = 0.6 MPa)^6^. Biological bone tissue (cortical and cancellous) is a highly inhomogeneous material with pronounced orientations of internal structures, leading to its anisotropic mechanical properties^6^. Biological bone tissue (cortical and cancellous) is a highly inhomogeneous material with pronounced orientations of internal structures, leading to its anisotropic mechanical properties. Moreover, these mechanical properties are inherently nonlinear. Describing such material properties using conventional methods is very demanding and impossible without adopting certain simplifications. Our FEM analyses modeled bone tissue as an inhomogeneous, isotropic, and elastoplastic material. The material properties of each element were determined depending on the density of bone tissue $\:\rho\:$. The density was determined depending on the degree of a gray color on CT scans of the distal end of the femur according to the relationship

$$\:\rho\:=1.54\cdot\:{\rho\:}_{CT}+0.0784$$

Where $\:{\rho\:}_{CT}$ is the density of the calibration sample^7^. The elastic modulus of elasticity E for both types of bone tissue (compact and cancellous) were determined using the relationships^8–10^. Where $\:{\rho\:}_{CT}$ is the density of the calibration sample^7^. The elastic modulus of elasticity E for both types of bone tissue (compact and cancellous) were determined using the relationships^8–10^.

$$\:\begin{array}{ccc}{E}^{k}=2065\cdot\:{\rho\:}^{3.09}&\:,&\:{\mu\:}^{k}=0.3\\\:{E}^{s}=1904\cdot\:{\rho\:}^{1.64}&\:,&\:{\mu\:}^{s}=0.3\end{array}$$

In the same way, yield stress $\:{\sigma\:}_{y}^{}$was determined as a function dependent on bone density according to

$$\:\begin{array}{ccc}{\sigma\:}_{y}^{k}=57.75\cdot\:{\rho\:}^{1.73}&\:pro&\:\rho\:\ge\:0.945\\\:{\sigma\:}_{y}^{s}=76.5\cdot\:{\rho\:}^{6.7}&\:pro&\:\rho\:<0.945\end{array}$$

**Fig. 1:**
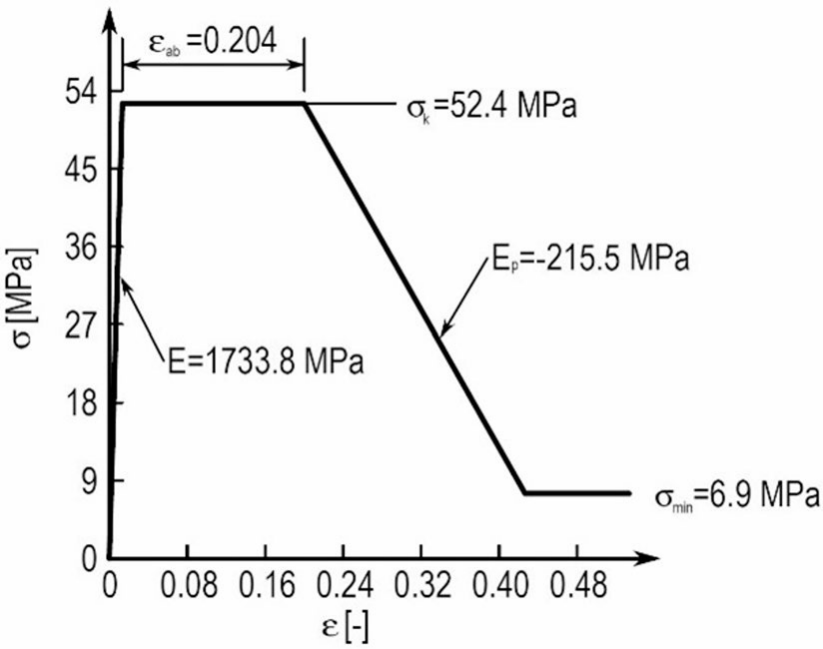
Specification of material properties: demonstration of the dependence of stress σ [MPa] on the strain **ε** [-] for bone tissue with a density of $\:{\uprho\:}$=0.945 [g/cm^3^].

In the computational analyses, bone tissue was also modeled as a material whose mechanical properties degraded after exceeding the load limit. This property is figuratively understood as a “degradation” of bone tissue^10^. These modeled properties are best illustrated by the graph in Fig. 1 presents the relationship between the stress σ and the strains ε of bone tissue^10^. These modeled properties are best illustrated by the graph in Fig. 1 presents the relationship between the stress σ and the strains ε of bone tissue. Individual values unambiguously describing the behavior of the material model when the yield strength exceeded σ_y_ was determined again depending on the density of bone tissue ρ according to the relationships.

$$\:\begin{array}{cc}{\sigma\:}_{min}=8.5\cdot\:{\rho\:}^{3.68}&\:{\varepsilon\:}_{ab}=0.258\cdot\:\rho\:-0.07\\\:{E}_{p}=-244\cdot\:{\rho\:}^{2.2}&\:{\varepsilon\:}_{bc}=\left|\frac{{\sigma\:}_{y}-{\sigma\:}_{min}}{{E}_{p}}+{\varepsilon\:}_{ab}\right|\end{array}$$

The hip bones were meshed using tetrahedral linear elements, where the global size of the elements in the bone tissue was 5 mm, and in the vicinity of the pubic symphysis, the mesh size was reduced to 1 mm. The pubic symphysis was meshed using hexahedral quadratic elements with a hybrid formulation with a global size of 0.81 mm. Numerical analyses were treated as a static nonlinear contact problem. The initial increment size was 0.05 mm, and the maximum increment size was 0.1 mm.

The transfer of forces through the interface between bone and symphysis was achieved in numerical models by *tie* constrains. A *tie* constrains is a *surface-to-surface* formulation that links surface nodes in contact with each other for the duration of the simulation. The coupling guarantees that the degree of freedom of nodes on the master surface is identical to the degree of freedom of nodes on the slave surface, i.e., the transfer of compressive and tensile forces. The interaction can be understood as “gluing” the two surfaces together. The acting force (reaction force on the hip joint) was placed to a reference point in the center of the left acetabulum. The distribution of force into the model was achieved using *distributed coupling*.

This connection distributes force from the reference node to the contact surface in the acetabulum. Forces from individual muscles located in the pelvic region and inserting on the hip and femur bones were distributed similarly, i.e., from the reference point, the applied force was distributed using *distributed coupling* to the area corresponding to the muscle attachment points.

The numerical model was designed so that displacement in all directions was prevented at the point of contact between the sacrum and the left hip bone, i.e., the left sacroiliac joint (SIJ). On the right side, contact between the hip bone and the sacrum at the right SIJ was modeled elastically, using a spring stiffness of k = 4,285 N.mm^−1^^11^. This stiffness corresponds approximately to the stiffness of a physiological SIJ.

The physiological pelvic model was loaded with an applied single force acting at the reference point of the right acetabulum. Its magnitude corresponds to the reaction force acting in the joint during the step phase when a person stands on the right leg, occurring at t = 0.74 s. The magnitudes of the individual components of the reaction force were: F_1_ = 852 N, F_2_ = 2,052 N, and F_3_ = − 1,042 N. The forces were adopted from the results of in vivo experimental measurements available online. The methodology of these experimental measurements was presented in^12^ At t = 0.74 s, the gait phase corresponding to single-leg stance is reached, at which point the loading of the hip joint and the pelvis is maximal.

**Fig. 2:**
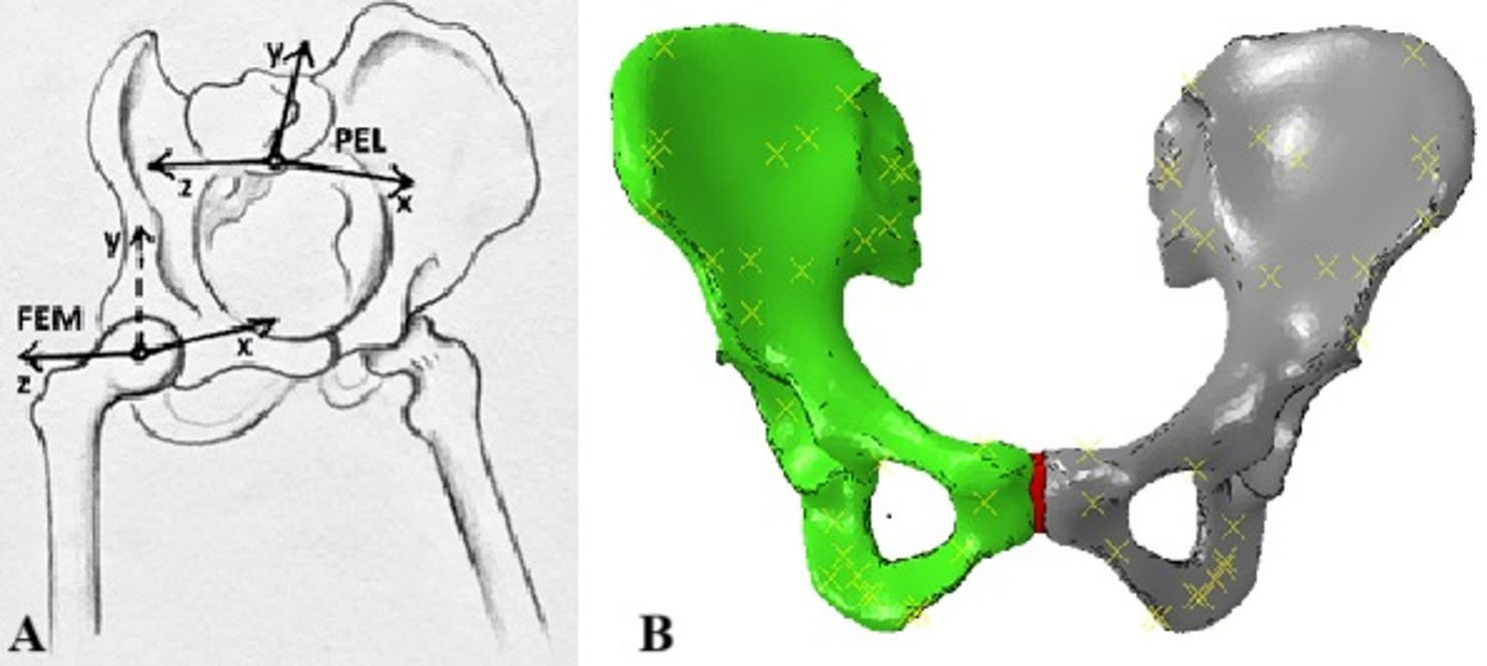
Coordinate system and places of action of muscles in the pelvic region: **A** – established coordinate system (FEM – head of femur, PEL – pelvis); **B** – demonstration of the position of the muscles acting on the pelvic model (yellow crosses).

The established coordinate system can be seen in Fig. 2A-B. The numerical model was further loaded with resultant forces representing individual muscles located in the pelvic region. The calculation of muscle forces was achieved using a 3D model of the human musculoskeletal system. The model had 23 degrees of freedom and contained 92 tendon-muscle actuators representing 76 muscles acting on the pelvis and lower limbs. Weights, moments of inertia, and positions of the center of gravity were given for individual segments in the model. The dynamic properties of the tendon–muscle complexes in the model were described using a Hill-type approach, which assumes that the resulting muscle force is a function of muscle length $\:{\:f}_{L}=\left({L}^{M}\right)$, rate of contraction $\:{f}_{v}=\left(\dot{{L}^{M}}\right)$ and degree of muscle activation $\:a\left(t\right)$ and also depends on the free length of the tendon and the angle of muscle pinnate. Using the inverse kinematic $\:{L}_{s\:\:}^{T}\alpha\:$ problem, kinematic attributes of the measured gait were assigned to the model; in the next step, the inverse dynamics procedure was applied, whereby the magnitudes of the reactions in individual joints during movement were calculated. The problem of the distribution of muscle forces for individual segments was solved using static optimization. The optimization criterion was.

1 $$\:min\sum\:{\left({a}_{i}\right)}^{2}$$

2 $$\:{Q}_{j}=\sum\:\left|{a}_{i}f\left({F}_{0,i}^{M},{L}_{i}^{M},{v}_{i}^{M}\right)\right|\cdot\:{r}_{i,j}$$

where $\:{a}_{i}$ is the muscle activation and takes values, $\:{a}_{i}0.01\le\:{a}_{i}\le\:1$, $\:{Q}_{j}$ are the reaction moments in the joint during movement, $\:{F}_{0,i}^{M}$ is the maximum isometric force of the muscle, $\:{L}_{i}^{M}$is the muscle length, $\:{v}_{i}^{M}$ is the speed of contraction of the muscle and $\:{r}_{i,j}\:$is the arm on which the respective muscle acts. The physiological parameters of the muscle were included in the calculation, and the optimization was solved using quadratic programming. Individual values of these forces are shown in Table 1, and their position is shown in Fig. 2A-B.

**Table 1:** The magnitude of muscle forces active when standing on one limb while walking normally.

Muscle	Force [N]	Muscle	Force [N]
*Adductor brevis*	0.00	*Gracilis*	0.00
*Adductor longus*	0.00	*Iliacue*	37.2
*Adductor magnus 1*	0.00	*Pectineus*	0.00
*Adductor magnus 2*	0.00	*Piriformis*	0.02
*Adductor magnus 3*	0.00	*Psoas major*	52.8
*Biceps femoris – long head*	0.02	*Quadratus femoris*	0.00
*Biceps femoris – short head*	33.2	*Rectus femoris*	43.5
*Gluteus maximus 1*	47.8	*Sartorius*	13.I
*Gluteus maximus 2*	27.IV	*Semimembranosus*	0.03
*Gluteus maximus 3*	0.00	*Semitendinosus*	0.01
*Gluteus medius 1*	620.6	*Tensor fasciae latae*	81.8
*Gluteus medius 2*	251.6		
*Gluteus medius 3*	197.9		
*Gluteus minimus 1*	71.5		
*Gluteus minimus 2*	76.4		
*Gluteus minimus 3*	77.5		

## Results

**Fig. 3:**
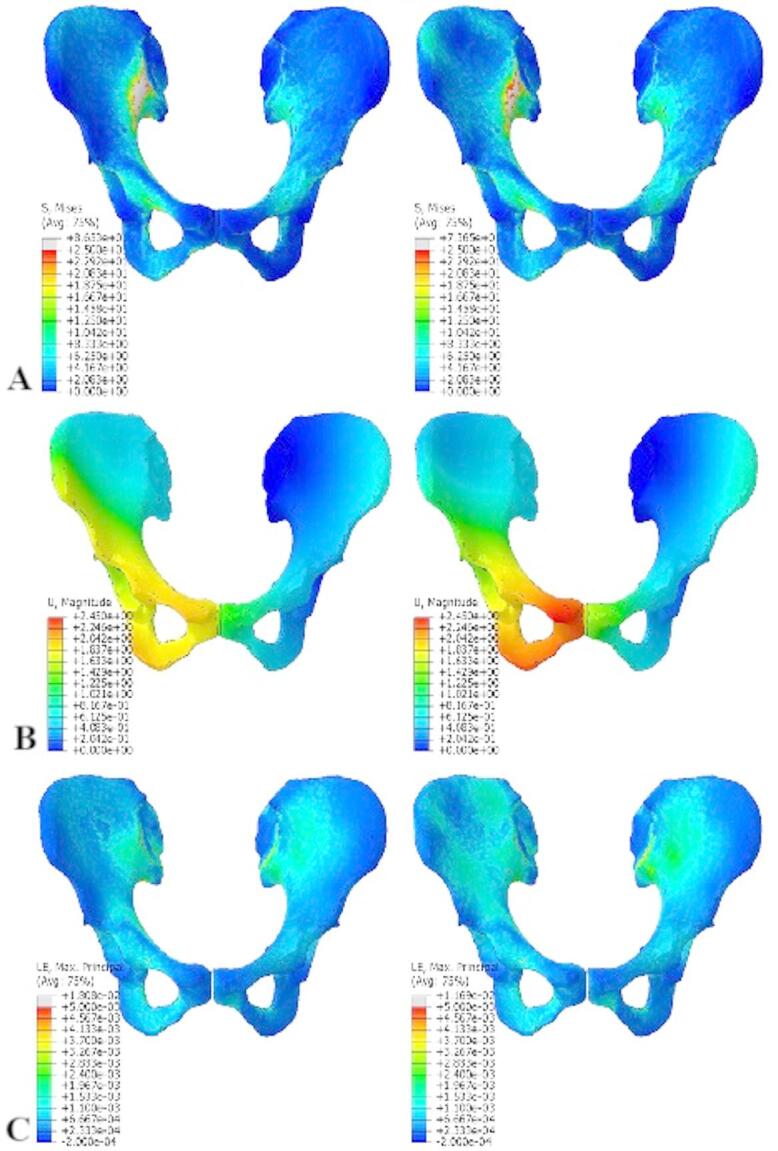
Results of FEM analyses for hip bones. There are results for the model loaded with the reaction force in the left column, and those for the model loaded with muscle forces in the right column: **A** – distribution of reduced stress σ_red_ [MPa]; **B** – distribution of displacements u [mm]; **C** – distribution of logarithmic strains **ε** [-].

**Fig. 4:**
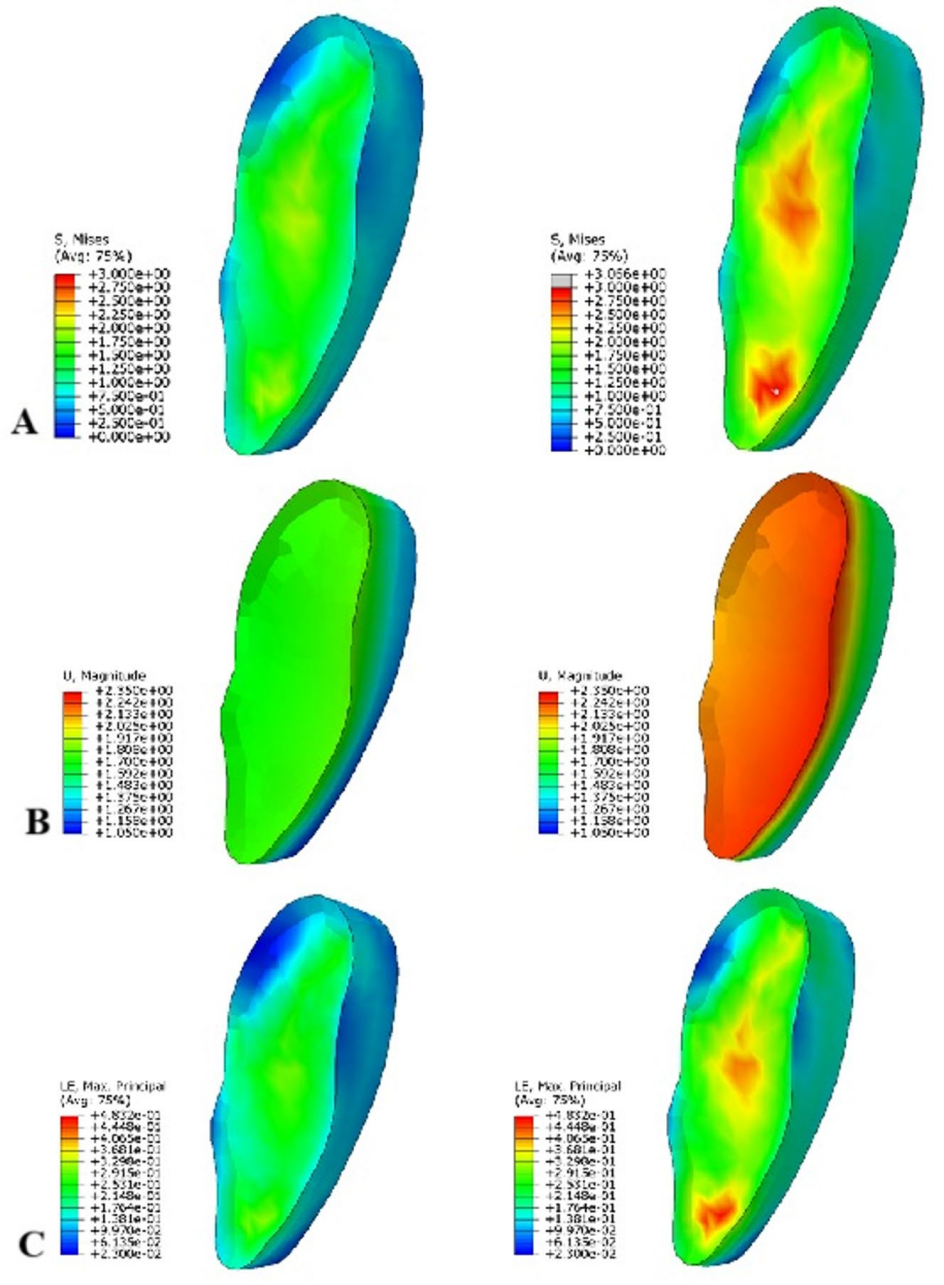
Results of FEM analyses performed on the pubic symphysis (anterior is on the right, posterior is on the left). There are the results for the model loaded with the reaction force in the left column, and those for the model loaded with muscle forces in the right column: **A** – distribution of reduced stress σ_red_ [MPa]; **B** – distribution of displacements u [mm]; **C** – distribution of logarithmic strains **ε** [-].

Our FEM analyses aimed to determine the stress, strains and displacements placed on bone tissue and the symphysis induced by external forces. The analyses were performed on a physiological pelvis model; the model was loaded with a single force corresponding to the reaction force in the hip joint. Furthermore, the model was loaded with forces from muscles that are attached on the pelvis. The criterion for the evaluation of static strength was the maximum value of the reduced stress σ_red_ [MPa], which was monitored in all components of the analysis model. The numerical analyses were created to allow stress solutions in bone tissue, even in plastic regions (by isotropic material hardening between the yield stress σ_y_ and the ultimate strength σ_m_). The results of the numerical analyses are clearly shown in Table 2; Figs. 3A-C and 4A-C.

**Table 2:** Overview of resulting values of stress σ_red_, displacement (u), and strains **ε**_max_,** ε**_min_ of the individual parts of the analysis model.

	σ_red_ [MPa]	u [mm]	ε_max_ [-]	ε_min_ [-]
	**Reaction force load in the joint**			
**Hip bones**	86.5	02.XI	1.81.10^−2^	−2.18.10^−2^
**Pubic symphysis**	02.XII	I.83	3.28.10^−1^	−3.95.10^−2^
	**Load with muscle forces**			
**Hip bones**	73.7	II.45	1.17.10^−2^	−1.90.10^−2^
**Pubic symphysis**	03.I	II.31	4.61.10^−1^	−5.49.10^−2^

### Hip bones

The results of numerical analyses of hip bones are shown in Table 2. The yield stress σ_y_ was slightly exceeded in the area of the right SIJ, where the right hip bone was fixed. The distribution and magnitude of reduced stress σ_red_ (Fig. 3A-C) showed that the highest stress concentration was, as expected, at the margin of the inferior public ligament (arcuate line). As expected, the magnitude of the reduced stress σ_red_ was larger in the model loaded only with the reaction force, where the bone load was 14.8% higher. On the other hand, the analyzed pelvic model loaded with forces from muscle acting on the pelvis had 13.8% less stiffness when loaded (Fig. 3A-C).

### Pubic symphysis

The pubic symphysis was more loaded in the pelvic model with forces from attached muscles, where the magnitude of reduced stress σ_red_ was greater by 29.5%. Of course, the size of pubic symphysis strains in this model was larger by 26.2%. The maximum strains of the pubic symphysis were localized to two places that are permanently loaded during physiological walking (Fig. 4A-C).

### The magnitude of the reaction force in SIJ

FEM analyses determined the magnitude of the force acting at the right-side SIJ. The components of the reaction forces were: F_x_^SI^ = − 381.5 N, F_y_^SI^ = 463.0 N, and F_z_^SI^ = − 829.6 N. The components of the force are given by the coordinate system shown in (Fig. 2A-B). The magnitude of the applied force $\:\left|\text{F}\right|=\:\sqrt{{F}_{x}^{SI\:2}+{F}_{y}^{SI\:2}+{F}_{z}^{SI\:2}}$ was $\:1023.8\:\text{N}.$.

## Discussion

### Morphology of the pubic symphysis and the interpubic cavity

The pubic symphysis is a secondary cartilaginous joint, syndesmosis, or amphiarthrosis^13^. The pubic symphysis is composed of a pair of articular surfaces (symphysial surface) on the pubic bones, an interpubic disc of fibrocartilage, and two main symphysial ligaments (inferior and superior). The joint allows very limited movement of approximately 0.5–1 mm^14^. The interpubic disc is a fibrocartilage connective tissue structure between the pubic bones, which may contain a cavity. The ligamentous structures of the pubic symphysis consist of four main symphysial ligaments^15^. The pubic symphysis in vivo is not rigid since it is directly and indirectly affected by several muscle groups, which makes it challenging to model the dynamic stabilizers of the anterior pelvic segment and the pubic symphysis. In our study, therefore, to simplify the model, we decided to include only those muscles that had the greatest impact in previous studies, i.e., gluteal muscles, pelvitrochanteric muscles, thigh muscles, and the iliopsoas muscle^6,16,17^. The model did not include abdominal and pelvic floor muscles for simplicity, but this does not mean we consider these muscles insignificant.

**Fig. 5:**
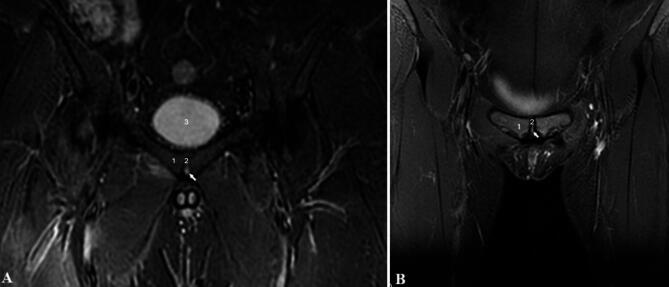
MR examination of the pubic symphysis (frontal plane TSET_2_ FAT SAT with a focus on the symphysial joint). The white arrows indicate the primary interpubic cavity in the inferior area of the interpubic disc, which strikingly correlated with the distribution of reduced stress seen in the FEM analysis: **A** – male pelvis, **B** – female pelvis. 1 - symphysis pubis, 2 - pubic symphysis midline, 3 - bladder.

The IPC can be divided into primary and secondary according to its origin. A primary cavity is defined as appearing around the second year postnatally and a secondary cavity as appearing in adulthood^2^. The primary IPCs resemble a lens in shape and have well defined contours^3^. The primary pubic cavity, which begins to appear around the second year of life^2^ at the end of the transition to bipedal walking, is associated with a biomechanical change in the load on the pubic symphysis during walking. The highest frequency of occurrence is in the youngest age group of adults and decreases with age. The primary cavity has precisely defined contours and contains fluid of unknown composition (Fig. 5A-B). Causes of the primary IPC forming are unknown and have not been described in any available literature yet. The term “secondary cavity” may be confusing as it does not meet the necessary criteria to be considered a true cavity. It is rather an oedematous lesion in the interpubic disc resulting from an underlying pathological process^4^. The secondary cavity is usually called a “cleft sign”^4,5,18,19^. The secondary cavity is poorly defined and irregularly shaped. Our findings show that the frequency of primary cavities in the age range from 20 to 40 years is about 10%; its incidence decreases with age in both men and women. Secondary cavities are found in about 30% of people, and the frequency increases with age in both men and women. A primary interpubic cavity is present in the anterior portion of the interpubic disc, within 2 mm from its anterior margin and the location of the cavity is more common in the middle and caudal part of the interpubic disc^3^. According to our FEM study, the area associated with the distribution of reduced stress (Fig. 4A-C), i.e., the middle and caudal parts of the interpubic disc, correlates very well with the frequency of occurrence of the primary cavity. It can therefore be assumed that the cavity helps the joint with load bearing during the transition to bipedal walking, i.e., at the time of high biomechanical load. The decrease in the incidence of the primary cavity with age can be explained by the adaptation of peri-symphysial stabilization structures and, thus, a reduced need for the primary cavity to facilitate load bearing. Unfortunately, verifying our hypothesis would require histological and cadaveric studies in children and younger individuals, where it would be possible to determine the contents and histological structure of symphysial cavities. Regrettably, such a study would encounter almost insurmountable legislative obstacles. However, this biomechanical study is an indirect confirmation of the hypothesis of the origin of the primary interpubic cavity. It can be assumed that the cavity, which is filled with liquid, serves to reduce the heavy load during the transition to bipedal walking. The subsequent appearance of a secondary interpubic cavity has a completely different biological basis, arising as a result of another pathological process affecting the symphysial joint^3^. According to our FEM study, the area associated with the distribution of reduced stress (Fig. 4A-C), i.e., the middle and caudal parts of the interpubic disc, correlates very well with the frequency of occurrence of the primary cavity. It can therefore be assumed that the cavity helps the joint with load bearing during the transition to bipedal walking, i.e., at the time of high biomechanical load. The decrease in the incidence of the primary cavity with age can be explained by the adaptation of peri-symphysial stabilization structures and, thus, a reduced need for the primary cavity to facilitate load bearing. Unfortunately, verifying our hypothesis would require histological and cadaveric studies in children and younger individuals, where it would be possible to determine the contents and histological structure of symphysial cavities. Regrettably, such a study would encounter almost insurmountable legislative obstacles. However, this biomechanical study is an indirect confirmation of the hypothesis of the origin of the primary interpubic cavity. It can be assumed that the cavity, which is filled with liquid, serves to reduce the heavy load during the transition to bipedal walking. The subsequent appearance of a secondary interpubic cavity has a completely different biological basis, arising as a result of another pathological process affecting the symphysial joint.

### Model and stability of symphysis fixation

Biomechanics of the pubic symphysis can be studied on anatomical specimens, but the type of preparation (fixed vs. fresh) negatively affects the mechanical properties of the ligamentous structures. Newer composite models use bone biomechanically equivalent to the physiological pelvis, but in the pubic symphysis area, they have vastly different properties, e.g., a rigid joint (gluing) or a connection with non-physiologically low stiffness, i.e., a foam or rubber strip. Using finite element models (FEM) avoids these problems by allowing simultaneous assessment of the influence of dynamic symphysis stabilizers, i.e., the influence of mechanical tension of muscle origins and insertions.

The dynamic load can be studied, using the standing model, while standing on both legs and while standing on one leg, which creates greater forces that are transmitted through the acetabulum onto the pubic symphysis. However, the modeling approaches differ from the biomechanical point of view, i.e., while standing on both legs, there is more mechanical stress on the pubic symphysis in the horizontal direction (in the direction of the x-axis), which is associated with tensile forces that prevent the pubic symphysis from “opening” up. The load while standing on only one leg is associated with higher stress on the pubic symphysis in the vertical direction, i.e., bending stress, i.e., stress in the direction of the y-axis. Sometimes, it is necessary to add a simulation of the rotation of the trunk (e.g., a combination of a heavy force through the acetabulum and a rotational moment, also through the acetabulum). This is the best way to show the rotational dislocations of the pubic symphysis caused by forced (violent) rotation of the hemipelvis caused by torsional forces; in the case of internal rotation, it is also associated with a compressive load on the pubic symphysis that can lead to its “closure,” i.e., symphyseal tilt^16^. This type of load in a FEM model was discussed in detail by Yao et al.^16,17^. This type of load in a FEM model was discussed in detail by Yao et al.^17,20^. Fixation can be done using standard pelvic 3.5 mm plates or angularly stable plates, a single plate, a combination of one cranial and one ventral plate, a combination of one cranial plate and ventral tensile cerclage on divergently inserted screws into the para-symphysial bone^21^, or dorsal plates with cranial “anchors”^22^. Fixation can be done using standard pelvic 3.5 mm plates or angularly stable plates, a single plate, a combination of one cranial and one ventral plate, a combination of one cranial plate and ventral tensile cerclage on divergently inserted screws into the para-symphysial bone^21^, or dorsal plates with cranial “anchors”^22^. The limitation of the study is that it was a pilot study and therefore the measurement was performed on only one model. We believe that it can be a template for further work. Furthermore, the results could be compared with a cadaveric study, which our study does not include.

## Conclusion

The pubic symphysis had a greater load in the pelvic model loaded by forces from the muscle groups, and, of course, the degree of symphysis deformation in this model was also greater. The primary interpubic cavity likely represents a biomechanical adaptation for load distribution during the early stages of bipedalism, with its location correlating with stress-dissipating regions. The maximum symphysis deformation was localized in two places, which are permanently loaded during physiological walking. It can therefore be assumed that the cavity helps the joint with load bearing during the transition to bipedal walking, i.e., at the time of high biomechanical load.

## Data Availability

Measurement data is available upon request.
